# The relationship between dietary quality and the local food environment differs according to level of educational attainment: A cross-sectional study

**DOI:** 10.1371/journal.pone.0183700

**Published:** 2017-08-25

**Authors:** Christina Vogel, Daniel Lewis, Georgia Ntani, Steven Cummins, Cyrus Cooper, Graham Moon, Janis Baird

**Affiliations:** 1 Medical Research Council Lifecourse Epidemiology Unit, University of Southampton, Southampton General Hospital Tremona Road, Southampton, United Kingdom; 2 Department of Social and Environmental Health Research, Faculty of Public Health & Policy, London School of Hygiene & Tropical Medicine, 15–17 Tavistock Place, London, United Kingdom; 3 NIHR Southampton Biomedical Research Centre, University Hospital Southampton NHS Foundation Trust and University of Southampton, Southampton, United Kingdom; 4 Geography and Environment, University of Southampton, Southampton, United Kingdom; TNO, NETHERLANDS

## Abstract

There is evidence that food outlet access differs according to level of neighbourhood deprivation but little is known about how individual circumstances affect associations between food outlet access and diet. This study explored the relationship between dietary quality and a measure of overall food environment, representing the balance between healthy and unhealthy food outlet access in individualised activity spaces. Furthermore, this study is the first to assess effect modification of level of educational attainment on this relationship. A total of 839 mothers with young children from Hampshire, United Kingdom (UK) completed a cross-sectional survey including a 20-item food frequency questionnaire to measure diet and questions about demographic characteristics and frequently visited locations including home, children’s centre, general practitioner, work, main food shop and physical activity location. Dietary information was used to calculate a standardised dietary quality score for each mother. Individualised activity spaces were produced by creating a 1000m buffer around frequently visited locations using ArcGIS. Cross-sectional observational food outlet data were overlaid onto activity spaces to derive an overall food environment score for each mother. These scores represented the balance between healthy and unhealthy food outlets using weightings to characterise the proportion of healthy or unhealthy foods sold in each outlet type. Food outlet access was dominated by the presence of unhealthy food outlets; only 1% of mothers were exposed to a healthy overall food environment in their daily activities. Level of educational attainment moderated the relationship between overall food environment and diet (mid vs low, p = 0.06; high vs low, p = 0.04). Adjusted stratified linear regression analyses showed poorer food environments were associated with better dietary quality among mothers with degrees (β = -0.02; 95%CI: -0.03, -0.001) and a tendency toward poorer dietary quality among mothers with low educational attainment, however this relationship was not statistically significant (β = 0.01; 95%CI: -0.01, 0.02). This study showed that unhealthy food outlets, like takeaways and convenience stores, dominated mothers’ food outlet access, and provides some empirical evidence to support the concept that individual characteristics, particularly educational attainment, are protective against exposure to unhealthy food environments. Improvements to the imbalance of healthy and unhealthy food outlets through planning restrictions could be important to reduce dietary inequalities.

## Introduction

Noncommunicable diseases (NCDs), such as cardiovascular diseases, claim 60% of deaths worldwide and the related social and economic costs are vast [1]. NCDs could be prevented by improving diets, especially among women of childbearing age [2]. The UK Scientific Advisory Committee on Nutrition has expressed concern over the poor diets of young women because of the impact they have on the short and long term health of their children, as well as their own health [2, 3]. Governments increasingly acknowledge that environments influence diet, and recognise the need to modify food environments to make healthier food choices easier, particularly for those from disadvantaged backgrounds [4, 5].

Reviews show there is good evidence for greater access to fast food outlets in neighbourhoods of higher deprivation, and some evidence that greater fast food outlet access is associated with poorer dietary behaviours [6, 7]. There is also some evidence for an association between increased access to stores selling healthy foods, such as supermarkets or grocery stores, and higher fruit and vegetable intake. There are, however, a number of methodological limitations that restrict attempts to draw clear conclusions about the relationship between food outlet access and dietary behaviours [6, 8]. Food access studies have tended to focus on a subset of food outlet types, typically supermarkets, and/or fast food outlets, and these restricted measures are likely to provide incomplete information about the food environment because they do not consider the full range of outlets accessible or the balance between the ‘healthy’ and ‘unhealthy’ outlets [9, 10]. A 2015 systematic review showed that studies assessing multiple types of food outlets were most consistently associated with obesity among adults [11] and there have been recommendations for future studies to include a wide range of outlets such as fruit and vegetable stores, health food stores and bakeries in addition to fast food outlets and supermarkets [6, 12]. The use of relative food outlet access measures which adjust for overall food outlet density has also been recommended because they provide a more complete picture of food access and may overcome the weak evidence for an association between food access and diet [13]. Additionally, measures that weight a wide range of food outlets according to the extent to which they sell healthy or unhealthy foods may offer further methodological advancements and provide more nuanced assessments of the relationship between the local food environment and diet [9]. No research assessing the relationship between diet and a comprehensive food environment measure has been performed in the UK.

The local food environment has traditionally been operationalised as exposure to food outlets in residential neighbourhoods [8]. In recent years, the approach has expanded to include exposures that more realistically represent an individual’s ‘activity space’—the locations that individuals typically visit in their daily lives including work, childcare or school [14]. Activity space measures have demonstrated additional explanatory power over residential measures alone in understanding the contextual correlates of health behaviours by accounting for individual daily mobility [15]. This methodological advancement has been demonstrated by the fact that food outlet exposure in activity spaces varies considerably from solely residential exposure [14, 16]. Moreover, a study among adults in Cambridgeshire found that greater exposure to takeaway outlets in home, work and commuting environments was associated with higher takeaway food intake; associations were stronger in work than home or commuting environments and there was evidence of a dose-response effect when the three areas were combined [17]. Further work assessing relationships between activity space exposures and dietary quality is needed.

The spatial accessibility of food outlets has been conceptualised as one component of the food environment that can influence diet [18], where greater access to outlets selling predominantly healthy foods or those selling mainly unhealthy foods can improve or worsen dietary choices respectively. This theoretical premise for an environment-health relationship proposes that access to food outlets can help or hinder an individual’s health independent of their individual-level risk factors, and may even exacerbate individual circumstances [19]. Previous research has shown that the environment localised to residential address is of greater importance to individuals of lower socioeconomic position who have lower mobility than to those who are more affluent [20]. However, evidence of how individual-level determinants of diet, such as educational attainment, modify the effect of environmental exposures on diet is limited [21]. Research from the UK has shown that the diets of disadvantaged women are more affected by less healthy supermarket environments than those of affluent women [22], and that associations between fast food outlet exposure and fast food intake are more pronounced for adults of low socioeconomic status than those of high socioeconomic status [23]. Level of educational attainment is the strongest predictor of young women’s diets [24] and shapes other socio-demographic markers including employment status, job type and income [25]. Understanding whether and how an individual’s educational attainment modifies the relationship between the local food environment and diet could help practitioners tailor person- and place-specific interventions aimed at dietary improvement.

The aims of this study were to: i) examine the relationship between individual dietary quality and a measure of overall food environment exposure from activity spaces in a sample of mothers with young children, and ii) assess the effect modification of mothers’ level of educational attainment on this relationship.

## Materials and methods

This study was cross-sectional, conducted in the UK and used follow-up participant data from the Southampton Initiative for Health (SIH), a complex community-based intervention study [26, 27], and environmental data from an observational survey. The study area covered the three council areas of the SIH (Southampton, Gosport and Havant) plus Eastleigh, Fareham and Portsmouth because participants reported food shopping or working in these surrounding areas. Southampton, Portsmouth and Havant have concentrated areas of high deprivation and are ranked 72^nd^, 76^th^ and 107^th^ respectively for deprivation out of the 326 local authorities in England (where 1^st^ is most deprived); Gosport, Eastleigh and Fareham are more affluent and are ranked 161^st^, 281^st^ and 315^th^ respectively [28]. More than 98% of the study area was classified as urban.

### Study sample

Participants were mothers who were pregnant or had a child under the age of five, and whose home residence was located within the study area. All mothers were recruited while attending Sure Start Children’s Centres [29] located in Southampton, Gosport and Havant. Between December 2010 and May 2011, 509 participants who were part of the SIH cohort completed questionnaires by telephone; an additional 412 were also recruited to enhance sample numbers and completed the questionnaire face-to-face. Mothers were asked for their home postcode and postcodes of locations frequently visited by walking, cycling, driving or public transport including workplace, Sure Start Children’s Centre, general practitioner, main supermarket and physical activity site. Mothers were also asked questions about their age, number of children, highest educational qualification obtained and whether they were in paid employment. Home postcode was used to determine each mothers’ level of neighbourhood deprivation (LSOA) using quintiles of the 2007 English Index of Deprivation income domain [30]. A 20-item food frequency questionnaire (FFQ) was used to assess dietary quality [31]. All study procedures, including acquiring written consent to participate from all mothers, were conducted according to the Declaration of Helsinki and were approved by the University of Southampton, Faculty of Medicine Ethics Committee.

### Dietary outcome–dietary quality score

The 20 item FFQ was statistically derived from a 100-item FFQ and contained foods consistent with the UK Department of Health’s recommendations for healthy eating and foods that contribute to noncommunicable diseases [24, 31]. Mothers were asked to indicate how often in the previous month they consumed each of the 20 foods (six point scale from ‘never’ to ‘once or more than once a day’). A dietary quality score was calculated for each mother by multiplying reported frequency of consumption for each FFQ item by its corresponding principal component coefficients and then summing the results [31]. The dietary scores were standardised to have a sample mean of zero and standard deviation (SD) of one. Diet scores calculated from this 20-item FFQ have correlated highly with scores from the 100-item FFQ (r = 0.94), and with red blood cell folate (r = 0.25) [31]. Positive scores characterised higher intakes of vegetables, vegetarian products and wholegrain bread and lower intakes of processed meats, crisps and sugar.

### Exposure measure–food environment scores

An initial list of 1682 retail and takeaway outlets and their postcodes within the study area was compiled in July and August 2010 using information from the Food Safety Register of the six local authorities in the study area. Information from on-line business directories (Yellow Pages and yell.com) was used to supplement local authority lists in an effort to obtain a complete picture of food outlet locations across the study area. Food outlets types were classified based on previous research in northern England [32]. Due to regional differences, some categories were informed by the Local Authority Enforcement Monitoring System (LAEMS)[33]. The first author of this manuscript completed all the ‘ground-truthing’ of the study area with assistance from four trained fieldworkers to confirm the existence and classification of stores between July 2010 and June 2011: 245 outlets were removed because they were no-longer present and 350 additional outlets were identified. A total of 1787 retail and takeaway food outlets were identified, including 606 supermarkets and convenience stores, 576 takeaway outlets and 80 fast food chains. Given the use of multiple sources and field-based ground-truthing, store identification should be comprehensive. It remains possible however that some outlets may have been missed. Using postcode information, 1787 outlets were geocoded to postcode centroid using Geoconvert and ArcGIS10.1 [34]. In the UK, postcode provides an abbreviated address that is unique to an average of 15 households; the maximum number of addresses in a postcode is 100 and one address receiving a large amount of mail can be allocated a single unique postcode. Less than 3% of locations were not matched in this study and this small number is most likely due to the overlap between the study period and the timing of the data used by Geoconvert (both 2011). In instances where addresses did not match, we used Google maps to zoom in on the street addresses and identify a proxy address.

**Fig 1:**
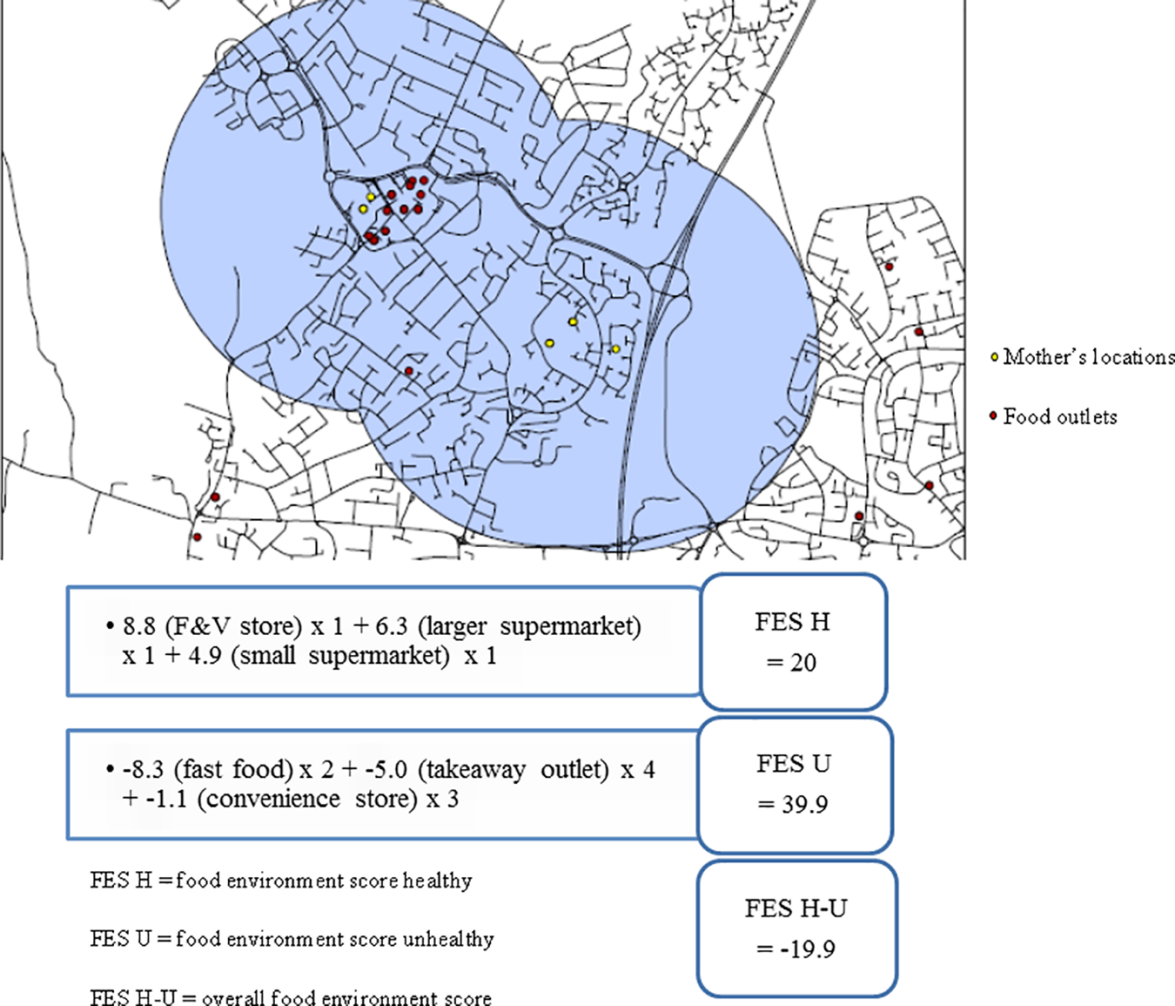
An example of a mother’s activity space with food outlets and food environment score calculation.

Individualised activity spaces were defined for each mother according to the total space covered by the set of 1000 meter (0.6 mile) buffers around home postcode centroid and around the postcode centroid of other frequently visited locations including main supermarket, Sure Start Children’s Centre, workplace, general practitioner and physical activity location. Mothers with fewer than two locations inside the study area were excluded. Buffers that overlapped were merged to avoid double counting of food outlets. The 1000 meter buffer distance created around the postcode centroid of each location corresponds to a 10–15 minute walk [35] and is the midpoint of buffers applied around home and work in previous activity space studies [17, 36]. Euclidean distance rather than road network was used because comparison studies have found little difference between the two measures at 1000 meters in urban areas [37]. The geographical area (km^2^) of each activity space was calculated to determine whether the size of mothers’ activity spaces differed by their level of educational attainment. Coordinates for all food outlet data were overlaid onto the individualised activity spaces and the number and type of outlets within each mother’s activity space was identified. An example of a mother’s activity space, their home and other locations and food outlets are shown in Fig 1.

Using these food outlet data, we calculated a score for each mother that represented the type and number of food outlets she was exposed to within her activity space [9]. These scores represented both spatial exposure to different types of food outlets and a proxy of the healthfulness of the in-store environment based on the availability of healthy and unhealthy foods in each type of outlet. Scores were calculated by: i) identifying the number of each type of food outlet within mothers’ activity spaces, and ii) multiplying the number of each food outlet by a weighting describing the relative availability of healthy and unhealthy foods within each type of food outlet. The weights were determined from a previous Delphi study where eight international leaders in food environment research rated different types of foods outlets from 10 (outlet encourages healthy eating) to -10 (outlet encourages unhealthy eating) and a mean rating for each food outlet type was calculated [9]. Food outlets with a positive mean weighting were classified as healthy food outlets and those with a negative mean weighting were classified as unhealthy food outlets. For example, the mean weighting of fruit and vegetables stores was 8.8 (indicating excellent availability of healthy foods) and the mean weighting of independent takeaways was -5.0 (indicating predominant availability of unhealthy foods). Table 1 shows the mean weightings for each type of food outlet determined from the earlier Delphi study and the corresponding category applied in the current study. Three food environment scores (FES) were created for each mother: i) food environment score healthy (FES H), calculated by summing the scores for outlets with positive weightings, ii) food environment score unhealthy (FES U), calculated by summing the scores outlets with negative weightings and iii) and overall food environment score (FES H-U) to measure each mothers relative access to healthy food outlets and unhealthy food outlets calculated by subtracting FES-U from FES-H. To illustrate the calculation of these scores, if a mother had 12 outlets in her activity space which included four takeaways, two fast food outlets, three convenience stores, a fruit and vegetable stores, a large supermarket and a small supermarket the calculation of her FES H, FES U and FES H-U are shown in Fig 1.

**Table 1:** Food outlet types and mean expert ratings from Thornton and Kavanagh[9] and the current study.

Mean rating score (SD)	Delphi study outlet type	Current study outlet type
**Healthy food outlets**		
8.8 (2.1)	Fruit and vegetable market	Farm shop
8.8 (2.1)	Fruit and vegetable store	Greengrocer
6.3 (2.9)	Supermarket–large chain	Premium/large supermarkets
5.4 (3.2)	Butcher	Butcher
5.3 (2.5)	Ethnic	‘World’ stores
5.0 (2.5)	Bakery–bread only	N/A
4.9 (2.7)	Supermarket–mid	Small supermarkets
4.4 (2.4)	Deli	Sandwich shops
4.3 (3.3)	Health	Health food stores
4.3 (2.9)	Convenience—fresh	N/A
3.3 (3.5)	Supermarket—discount	Discount supermarkets
0.8 (1.9)	Bakery–mixed	Bakery
**Unhealthy food outlets**		
-1.1 (4.1)	Convenience–non fresh	Convenience/petrol stores
-1.1 (2.3)	Takeaway–food court	N/A
-1.6 (2.4)	Takeaway–(Asian/Indian)	Chinese/Indian takeaways
-5.0 (0.9)	Takeaway–independent	Fish & chips/Other takeaways
-5.0 (3.6)	Other–miscellaneous	Newsagents/confectioners
-8.3 (1.6)	Takeaway–major chain	Fast food outlets

### Statistical analyses

Sensitivity analyses were completed to assess differences between the demographic characteristics of mothers included in this study and those excluded, and to detect differences between the two survey completion methods by using t-test for age at interview and chi-squared test for number of children, educational attainment and neighbourhood deprivation. Descriptive statistical analyses were also performed to explore the trend across three levels of educational attainment (low: school qualifications up to 16 years of age, mid: advanced level school qualifications/diploma, and high: tertiary degree), for mothers’ dietary quality, age, and employment status (employed or unemployed) using regression analysis. Trend across education levels for number of children, neighbourhood deprivation, number of frequently visited locations, activity space area (km^2^), road network distance to main supermarket (km), FES H, FES U, and FES H-U were assessed using Spearman test for trend.

To address the first research question, linear regression analysis was used to assess the relationship between mother’s dietary quality and the overall food environment score (FES H-U). To address the second research question, an interaction term for educational attainment and overall food environment score was added to the regression model to determine the modification effect of educational attainment. Stratified analyses were conducted to examine the association between the overall food environment and dietary quality among the three levels of educational attainment separately. Adjustments were made for covariates that were independently associated with dietary quality including age, number of children and level of neighbourhood deprivation. Employment status and number of frequently visited locations reported were also added to the regression models as potential confounders. Area of activity space and total number food outlets within activity space were not associated with dietary quality in the adjusted regression models and were removed from these analyses. While previous studies assessing the relationship between spatial access to food outlets and diet have used multilevel regression models, this approach was not appropriate for the current study because the dietary outcome was not clustered. All statistical analyses were completed using Stata statistical software package version 13.0.[38]

## Results

### Mothers’ characteristics

Out of the 921 mothers who participated in the SIH follow-up survey, a total of 839 (91%) mothers provided at least two frequently visited locations within the study area, and had complete information on educational attainment. Sensitivity analyses showed no difference in the number of children, highest educational attainment or neighbourhood deprivation between those included and those excluded from the study (all p≥0.1), however those mothers not included in this study were slightly older (p = 0.03). Further sensitivity analyses revealed some differences between the two groups of participants in the follow-up survey: mothers who completed the survey face-to-face were younger (p<0.001), more likely to have only one child (p<0.001), had lower levels of educational attainment (p = 0.03) and lived in more deprived neighbourhoods (p = 0.02) than mothers in the cohort who completed the follow-up survey by telephone. The additional cross-sectional sample bolstered the numbers of more disadvantaged mothers and combining both groups provided a sample with representation from across the socioeconomic spectrum. All participant information was treated entirely as a cross sectional sample.

Table 2 presents the characteristics of the 839 mothers that reported their educational attainment by the three education groups. A third of mothers (37%) had no educational qualifications beyond 16 years of age (low educational attainment). Mothers with low educational attainment were younger, had more children, tended to live in more deprived neighbourhoods (all p<0.001) and were less likely to be in paid employment (p = 0.002) than mothers with higher attainment. The mean dietary quality score for mothers with low educational attainment was significantly lower than that of mothers with higher educational attainment (p<0.001). Mothers with low educational attainment had a mean dietary quality score approximately one standard deviation lower than those with high educational attainment which is, for example, equivalent to eating salad vegetables up to six times less often, and crisps up to six times more often a week.

**Table 2:** Characteristics of mothers across three levels of educational attainment.

	All	Low education	Mid education	High education	
		(≤GCSE)		(Degree)	
	n = 839	n = 307	n = 298	n = 234	
	Mean (SD)				p-value
**Dietary quality score**	-0.01 (1.00)	-0.44 (0.97)	-0.03 (0.89)	0.57 (0.88)	<0.001^b^
**Age at interview**	32 (6)	31 (6)	32 (6)	34 (5)	<0.001^b^
		**n**^**a**^ **(%)**			
**Number of children**					<0.001^c^
Pregnant	5 (1)	1 (0)	4 (1)	0 (0)	
1	337 (40)	108 (35)	114 (38)	115 (49)	
2	337 (40)	114 (37)	130 (44)	93 (40)	
3	119 (14)	57 (19)	40 (13)	22 (9)	
4+	40 (5)	27 (9)	10 (3)	3 (1)	
**Neighbourhood deprivation**			)		<0.001^c^
Most deprived	171 (21)	92 (31)	65 (23)	14 (6)	
2	170 (21)	77 (26)	58 (20)	35 (16)	
3	239 (29)	75 (25)	87 (30)	77 (34	
4	115 (14)	28 (9)	40 (14)	47 (21)	
Least deprived	119 (15)	28 (9)	38 (13	53 (23)	
**Paid employment**					0.002^b^
No	501(60)	207 (67)	166 (56)	128 (55)	
Yes	338 (40)	100 (33)	132 (44)	106 (45)	

^a^Percentages may not add up to 100% due to rounding

^b^Regression test for trend

^c^Spearman test for trend

### Mothers’ activity spaces and food environment scores

The median number of locations frequently visited by mothers in the study was four (Interquartile range (IQR): 4, 5). The smallest number of locations reported was two (0.2%) and the greatest was six (9%). All mothers provided information about their home and Sure Start Children’s Centre; most mothers reported the location of their main food store and general practitioner practice (frequently visited by pregnant women and families with young children) (both 94%). Approximately one third of mothers (30%) reported a physical activity location other than home and 33% of mothers reported a work location that was different from home. Spearman test for trend showed the number of locations reported by mothers did not differ according to their level of educational attainment (p = 0.9, Table 3). The median geographical area of activity spaces was ten square kilometres (IQR: 8, 12). The smallest area covered four square kilometres and the largest area was 18 square kilometres. Spearman test for trend showed mothers with higher educational attainment had significantly larger activity spaces (p = 0.05, Table 3). The median number of total food outlets in mothers’ activity spaces was 71 (IQR: 52, 106), where the smallest number of total outlets was 7 and the largest was 284. Mothers with higher educational attainment had significantly more total food outlets than mothers with low attainment levels (p = 0.001, Table 3).

**Table 3:** Activity space and food outlet access measures across three levels of educational attainment.

	All	Low education	Mid education	High education	r^a^	p-value
		(≤GCSE)		(Degree)		
	n = 839	n = 307	n = 298	n = 234		
	Median (IQR)					
**Number of locations**	4 (4, 5)	4 (4, 5)	4 (4, 5)	4 (4, 5)	0.004	0.9
**Activity space area (km**^**2**^**)**	10 (8, 12)	9 (8, 11)	10 (8, 12)	10 (8, 12)	0.07	0.05
**Total number of outlets in activity space**	71 (52, 106)	68 (52, 95)	69 (49, 100)	85 (55, 123)	0.11	0.001
**Network distance to main supermarket (km)**	3.9 (2.3, 6.1)	3.5 (2.3, 5.5)	4.2 (2.5, 6.2)	3.9 (2.1, 6.4)	0.13	0.1
**Healthy FES**	98 (69, 138)	94 (65, 127)	92 (66, 129)	115 (72, 166)	0.13	0.0002
**Unhealthy FES**	173 (121, 250)	165 (119, 229)	166 (117, 236)	199 (134, 302)	0.10	0.003
**Overall FES (H-U)**	-78 (-117, -45)	-74 (-107, -45)	-77 (-111, -42)	-85 (-134, -49)	-0.06	0.08

^a^Spearman correlation coefficient to test for trend

The median overall food environment score (FES H-U) was -78, ranging from -387 to 31 (IQR: -117, -45). Almost all mothers (99%, n = 829) had negative overall food environment scores indicating greater exposure to food outlets selling predominantly unhealthy foods than to those selling mainly healthy foods in their activity spaces. The median healthy food environment score (FES H) was 98, with scores ranging from 12 to 445 (IQR: 69, 138). The median unhealthy food environment score (FES U) was 173, with a range from 9 to 810 (IQR: 121, 250). The median of both the healthy food environment score (FES H) and the unhealthy food environment score (FES U) were significantly greater for mothers with high educational attainment than for other mothers (r_spearman_ = 0.13, p = 0.0002 and r_spearman_ = 0.10, p = 0.003 respectively, Table 3). Due to the domination of outlets selling mainly unhealthy foods, mothers with degrees showed a tendency toward lower overall food environment scores than mothers with lower educational attainment (FES H-U) (r_spearman_ = -0.06, p = 0.08, Table 3).

### The relationship between activity space food environment scores and dietary quality

Univariate regression analyses showed that lower overall food environment scores were associated with better dietary quality (β = -0.01SD/ 10 environment score units; 95%CI: -0.02, -0.001, p = 0.03), indicating that mothers’ who were exposed to a higher proportion of unhealthy food outlets than healthy food outlets had healthier dietary patterns. In the multivariable model, adjusting for the potentially confounding variables of age, educational attainment, number of children, neighbourhood deprivation, employment status and number of frequently visited locations provided the relationship weakened slightly (β = -0.01; 95%CI: -0.02, 0.00, p = 0.06).

**Fig 2:**
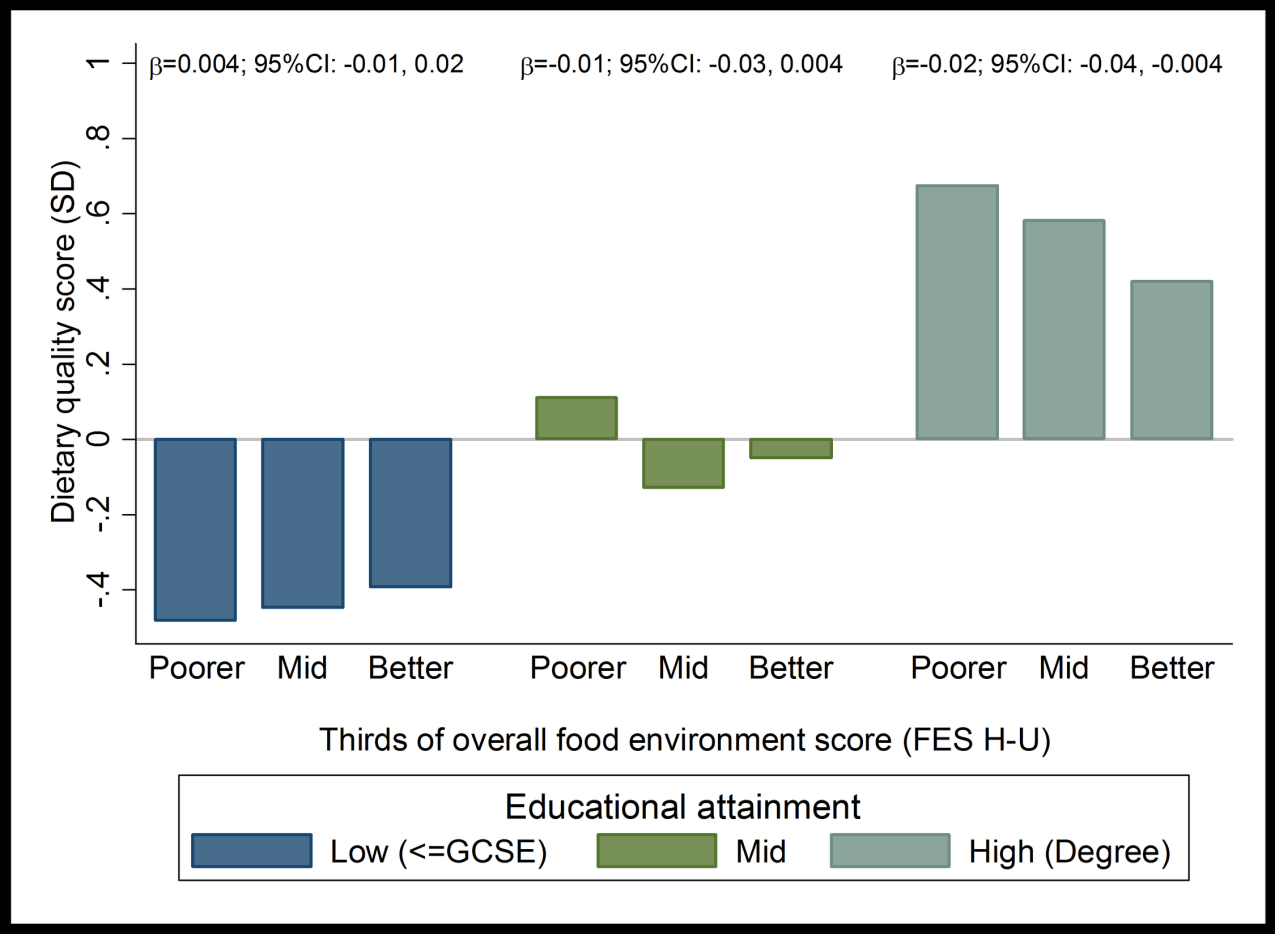
Thirds of food environment score (FES U-H) by mothers’ dietary quality according to their level of educational attainment.

Fig 2 illustrates the relationship between mothers’ dietary quality and overall food environment score according to level of educational attainment and indicates a difference in the direction of the association across the three levels; indicating a positive relationship among mothers with low educational attainment, no clear pattern among mothers with mid educational attainment and an inverse relationship among mothers with degrees. This difference was supported by evidence of an interaction between educational attainment and overall food environment score, with a differences observed between low and high educational attainment (p = 0.04) and between low and mid educational attainment (p = 0.06). Stratified regression models confirmed differences in the direction of exposure-diet relationships between mothers with low and those with higher educational attainment. Among mothers with high and mid educational attainment, poorer food environments were related to better dietary quality (β = -0.02 SD/ 10 environment score units; 95%CI: -0.04, -0.004 and β = -0.01; 95%CI: -0.03, 0.004 respectively, Table 4). However, among mothers with low educational attainment, there was a trend in the opposite direction, where mothers exposed to healthier food environments tended to have better dietary quality, although the relationship was not significant (β = 0.004; 95%CI: -0.01, 0.02). In models adjusted for confounding variables, the inverse relationships among mothers with high educational attainment remained robust (β = -0.02; 95%CI: -0.03, -0.001).

**Table 4:** Three linear regression models showing the association between overall food environment and mothers’ dietary quality among women of a) low, b) mid, and c) high education, and separate regression models testing for interaction between education and environment.

Among women of:	Dietary quality score		
	(SD/ 10 environment score units)		
	β (95% CI), p-value		
	*Unadjusted model*	*Adjusted model*^*a*^	*Interaction*^*(FES and diet)*^
**Low education** (≤GCSE) n = 307	0.004 (-0.01, 0.02),	0.01 (-0.01, 0.02),	low & mid β = -0.02, p = 0.06
	0.6	0.3	
**Mid education** n = 298	-0.01 (-0.03, 0.004),	-0.02 (-0.03,0.002),	
			low & high β = -0.02, p = 0.04
	0.1	0.08	
**High education** (Degree) n = 234	-0.02 (-0.04, -0.004),	-0.02 (-0.03, -0.001),	
	0.02	0.04	

^a^ Confounding variables: age, number of children, neighbourhood deprivation, employment status, and number of reported locations. Of the full sample of 839 mothers, 17 did not provide information on all confounding variables therefore the adjusted models included 300 mothers with low educational attainment, 288 with mid educational attainment and 225 with high educational attainment.

## Discussion

The findings of this study showed that poorer overall food environment scores were associated with better dietary quality among mothers with degrees but tended towards an association with poorer dietary quality among mothers with low educational attainment. Although these findings should be viewed with caution as not all associations were significant, they provide some indication that place-health relationships, particularly food outlet access-diet relationships, may differ according to level of educational attainment.

The significant difference in the direction of the association between overall food outlet exposure and dietary quality between women with low and high educational attainment provides some empirical evidence to support the concept that the accessibility of physical resources, which can help or hinder an individual’s health, can be exacerbated by individual circumstances. Mothers with degrees were, on average, exposed to the least healthy food environments, and yet had the best quality diets. This relationship likely results from these mothers being exposed to a greater number of food outlets than other mothers because a disproportionate number of all food outlets were rated as being unhealthy. It may be that mothers who are highly educated and highly health conscious work in and/or shop at supermarkets located in areas of high commercial density which increases their overall exposure to food retail. The growing market share of discount supermarkets such as Lidl and Aldi, and the rapid growth of small supermarkets, has seen the number of supermarkets in city centres and high streets increase over the last decade [39, 40]. We speculate that some highly educated women choose to shop in these locations for convenience or budgeting reasons and that, despite exposure to high numbers of unhealthy food outlets, visiting areas of high commercial density may enhance behavioural and health outcomes through mechanisms of increased employment and increased concentration of community services and resources [41]. This suggestion is consistent with previous research in the US which found that increased exposure to fast food outlets was linked to lower odds of obesity [42]. The researchers also found that greater exposure to banks was associated with lower odds of obesity and concluded that both fast food outlets and banks were co-located in areas of high commercial density.

The inverse relationship observed among mothers with degrees may also be attributed to these women having greater financial and psychological resources which enable them to make healthy food choices despite being exposed to poorer food environments. In this study, higher rates of paid employment were reported by mothers with higher educational attainment and it has been suggested that education is a determinant of other markers of socioeconomic position such as employment status, job role and income level acting through an increased sense of autonomy and critical thinking [25, 43]. The greater levels of agency and sense of control over food choices exhibited by women with higher education levels [44, 45] is likely to help them navigate complex and unhealthy food environments.

Even though there was no difference in the number of reported locations across the three educational groups, mothers with low educational attainment had smaller activity spaces. This finding is consistent with research from the US and is likely to reflect the greater mobility of individuals that are more affluent [16]. The smaller activity space of mothers with low educational attainment indicates the co-location of frequently visited locations and suggests that environmental exposures localised to residential address are more important for those with low education levels [46]. Even though the results of this study showed no significant relationship between overall food environment and dietary quality among mothers with low education, the reliance on residential area may have important implications for future interventions. Mothers in this study with low educational attainment were more likely to live in disadvantaged neighbourhoods and there is good evidence that takeaway and fast food outlets are more prevalent in more deprived neighbourhoods [6]. A recent study in Norfolk, UK revealed that the number of takeaway outlets increased between 1990 and 2008, and that the growth was significantly greater in more deprived neighbourhoods [47]. This increase in socioeconomic disparities in food outlet access suggests that action by governments to regulate the growth and abundance in takeaway and fast food outlets is likely to be of greatest benefit to those from disadvantaged backgrounds and who have the poorest quality diet [48].

Our results revealed that only 1% of mothers were exposed to a greater proportion of healthy than unhealthy food outlets in their activity spaces, indicating an overwhelming presence of outlets selling predominantly unhealthy food. Examining retail investment patterns to identify areas where commercial activity in unhealthy food outlets is disproportionately greater than that for healthy food outlets is important to improve public health. Areas of high deprivation may be seen as undesirable to some retailers due to high crime rates or lack of clientele, while other retailers may be more willing to trade in these areas because of low rent and cheap labour [49]. Zoning or licencing policies or incentives for healthy food retailers, such as fruit and vegetable stores, to open could improve the imbalance in healthy and unhealthy food outlets identified in this study. Financial constraints on local councils could hinder action, however, building evidence from natural experiments and advocating for central government support could enable councils to take action [50]. Further intervention research to explore the effects of increasing access to healthy specialty stores such as fruit and vegetables stores is needed to improve understanding of their potential beneficial effects on health [51].

### Strengths and limitations

A strength of this study was the use of an overall food environment score that represented both spatial exposure to different types of food outlets and considered a proxy of the availability of healthy and unhealthy foods within food outlets by weighting different types of outlets. These scores provide a more nuanced assessment than count measures because they discriminate between the availability of healthy and unhealthy foods within different outlet types. The use of activity spaces was also a strength because they represent the area mothers’ were exposed to while conducting their daily activities. In addition, the temporal connection between the food outlet and participant surveys increases confidence in the findings of this study and the use of a standardised dietary quality score provides a more robust outcome measure than brief dietary measures frequently used in food environment research [12, 52]. This study is also one of few to assess whether educational attainment modifies the relationship between the local food environment and dietary quality.

A limitation of this study is the cross-sectional and observational design which cannot infer causality of the associations observed. Moreover, the calculation of activity spaces assumed that mothers visited each of the reported locations equally because space and time adjustments were not made [20]. The use of GPS technology could have allowed for a more accurate measure of food environment exposure. The use of GPS technology remains in its infancy and poses a number of difficulties that can introduce bias including signal loss, delay in acquiring satellite signal, precision of the device, battery power, or participants forgetting to switch the device on [53]. This study provides a proxy measure of the relative availability of healthy and unhealthy foods within different types of food outlets. An in-store audit measuring the availability, cost, quality and promotion of healthy and unhealthy foods would need to be completed to accurately quantify the overall healthfulness of each outlet and could be considered in future research. Further research to confirm the moderation effect of educational attainment in larger samples and in different areas is also warranted.

## Conclusion

This study assessed differences in the relationship between food outlet access and diet according to level of educational attainment. A moderation effect was observed: poorer food environments were associated with better dietary quality among mothers with degrees and showed a tendency toward poorer dietary behaviours among mothers with low educational attainment, though this relationship was not significant. Furthermore, food outlet access was characterised by an overwhelming presence of unhealthy food outlets; only 1% were exposed to a higher proportion of healthy than unhealthy food outlets in their day-to-day activities. There is a need for local authorities to improve the balance of healthy and unhealthy local food retailing through planning restrictions or licensing initiatives and to build the evidence for action from natural experiments.

## Supporting information

S1 Table STROBE statement. (DOC)

## Abbreviations

FES: Food environment score
FES H: Food environment score for healthy food outlets
FES U: Food environment score for unhealthy food outlets
FES H-U: Overall food environment score
FFQ: Food Frequency Questionnaire
IQR: Interquartile Range
LSOA: Lower Super Output Area (small administrative boundary)
NCD: Noncommunicable Diseases
SIH: Southampton Initiative for Health
