# Supplementary material for: The relationship between dietary quality and the local food environment differs according to level of educational attainment: A cross-sectional study
Source: PLoS One. 2017 Aug 25;12(8):e0183700. doi: 10.1371/journal.pone.0183700 (PMC5571951; doi:10.1371/journal.pone.0183700)
Supplement: S1 Table — (DOC) [file pone.0183700.s001.doc]

S1 Table. STROBE Statement—Checklist of items that should be included in reports of ***cross-sectional studies***

|  | Item No | Recommendation |
| --- | --- | --- |
| **Title and abstract** | 1 | (*a*) Indicate the study’s design with a commonly used term in the title or the abstract **Abstract - methods** |
|  |  | (*b*) Provide in the abstract an informative and balanced summary of what was done and what was found  **Abstract – Methods and results** |
| Introduction | | |
| Background/rationale | 2 | Explain the scientific background and rationale for the investigation being reported  **Pages 3-5** |
| Objectives | 3 | State specific objectives, including any pre-specified hypotheses  **Page 6 - para 1** |
| Methods | | |
| Study design | 4 | Present key elements of study design early in the paper  **Abstract – methods section**  **Methods, page 6 – para 2** |
| Setting | 5 | Describe the setting, locations, and relevant dates, including periods of recruitment, exposure, follow-up, and data collection  **Page 6 – para 2 and para 3** |
| Participants | 6 | (*a*) Give the eligibility criteria, and the sources and methods of selection of participants  **Page 6 – para 3, page 7 – para 1** |
| Variables | 7 | Clearly define all outcomes, exposures, predictors, potential confounders, and effect modifiers. Give diagnostic criteria, if applicable  **page 7 – para 2, page 8 to page 10** |
| Data sources/ measurement | 8* | For each variable of interest, give sources of data and details of methods of assessment (measurement). Describe comparability of assessment methods if there is more than one group  **Page 6 – para 3, page 7 – para 1 and 2, page 8 – para 1, page 9 – para 1** |
| Bias | 9 | Describe any efforts to address potential sources of bias  **Page 5 – para 3, page 12 para 2, page 13 – para 1, page 21 – para 3** |
| Study size | 10 | Explain how the study size was arrived at  **Page 6 – para 3, page 7 – para 1, page 12 – para 2** |
| Quantitative variables | 11 | Explain how quantitative variables were handled in the analyses. If applicable, describe which groupings were chosen and why  **Pages 8 to 11** |
| Statistical methods | 12 | (*a*) Describe all statistical methods, including those used to control for confounding  **Pages 11, page 12 –para1** |
|  |  | (*b*) Describe any methods used to examine subgroups and interactions  **Page 12 – para 1** |
|  |  | (*c*) Explain how missing data were addressed  **N/A** |
|  |  | (*d*) If applicable, describe analytical methods taking account of sampling strategy  **N/A** |
|  |  | (*e*) Describe any sensitivity analyses  **Page 11** |
| Results | | |
| Participants | 13* | (a) Report numbers of individuals at each stage of study—eg numbers potentially eligible, examined for eligibility, confirmed eligible, included in the study, completing follow-up, and analysed  **Page 12 – para 2,page 13, Table 2** |
|  |  | (b) Give reasons for non-participation at each stage  **Page 12 – para 2** |
|  |  | (c) Consider use of a flow diagram  **N/A** |
| Descriptive data | 14* | (a) Give characteristics of study participants (eg demographic, clinical, social) and information on exposures and potential confounders  **Table 2, page 12 – para 1** |
|  |  | (b) Indicate number of participants with missing data for each variable of interest  **Table 2; Table 3; Table 4** |
| Outcome data | 15* | Report numbers of outcome events or summary measures  **Table 2, Table 3, pages 13 to 15** |
| Main results | 16 | (*a*) Give unadjusted estimates and, if applicable, confounder-adjusted estimates and their precision (eg, 95% confidence interval). Make clear which confounders were adjusted for and why they were included  **Table 4, pages 16 to 17** |
|  |  | (*b*) Report category boundaries when continuous variables were categorized  **Page 11, Table 2** |
|  |  | (*c*) If relevant, consider translating estimates of relative risk into absolute risk for a meaningful time period  **Dietary score quantified on page 13** |
| Other analyses | 17 | Report other analyses done—eg analyses of subgroups and interactions, and sensitivity analyses  **Tables 2-4, page 13 – para 2, pages 15 to 17** |
| Discussion | | |
| Key results | 18 | Summarise key results with reference to study objectives  **Page 18 – para 1 and 2** |
| Limitations | 19 | Discuss limitations of the study, taking into account sources of potential bias or imprecision. Discuss both direction and magnitude of any potential bias  **Page 21 – para 2 and 3** |
| Interpretation | 20 | Give a cautious overall interpretation of results considering objectives, limitations, multiplicity of analyses, results from similar studies, and other relevant evidence  **Page 18 – para 1, page 22 – para 2** |
| Generalisability | 21 | Discuss the generalisability (external validity) of the study results  **Page 21 – paras 2 and 3** |
| Other information | | |
| Funding | 22 | Give the source of funding and the role of the funders for the present study and, if applicable, for the original study on which the present article is based  **Funding disclosures** |

*Give information separately for exposed and unexposed groups.

**Note:** An Explanation and Elaboration article discusses each checklist item and gives methodological background and available on the Web sites of PLoS Medicine at http://www.plosmedicine.org/, Annals of Internal Medicine at http://www.annals.org/, and Epidemiology at http://www.epidem.com/). Information on the STROBE Initiative is available at www.strobe-statement.org.
